# Antibiotic Treatment during Pregnancy Alters Offspring Gut Microbiota in a Sex-Dependent Manner

**DOI:** 10.3390/biomedicines10051042

**Published:** 2022-04-30

**Authors:** Abdullah M. Madany, Heather K. Hughes, Paul Ashwood

**Affiliations:** 1 Department of Psychiatry and Behavioral Sciences, University of California Davis, Sacramento, CA 95616, USA; ammadany@ucdavis.edu; 2 The M.I.N.D. Institute, University of California Davis, Sacramento, CA 95817, USA; hkhughes@ucdavis.edu; 3 Department of Medical Microbiology and Immunology, University of California, Davis, CA 95616, USA

**Keywords:** antibiotics, gut microbiota, dysbiosis, autism spectrum disorder (ASD), lipopolysaccharide (LPS), *Lactobacillus*, inflammatory cytokines, interleukin (IL)-17, metabolic pathways

## Abstract

This study investigated the effect of antibiotics administered to pregnant dams on offspring gut microbiome composition and metabolic capabilities, and how these changes in the microbiota may influence their immune responses in both the periphery and the brain. We orally administered a broad-spectrum antibiotic (ABX) cocktail consisting of vancomycin 0.5 mg/mL, ampicillin 1 mg/mL, and neomycin 1 mg/mL to pregnant dams during late gestation through birth. Bacterial DNA was extracted from offspring fecal samples, and 16S ribosomal RNA gene was sequenced by Illumina, followed by analysis of gut microbiota composition and PICRUSt prediction. Serum and brain tissue cytokine levels were analyzed by Luminex. Our results indicate that the ABX-cocktail led to significant diversity and taxonomic changes to the offspring’s gut microbiome. In addition, the predicted KEGG and MetaCyc pathways were significantly altered in the offspring. Finally, there were decreased innate inflammatory cytokines and chemokines and interleukin (IL)-17 seen in the brains of ABX-cocktail offspring in response to lipopolysaccharide (LPS) immune challenge. Our results suggest that maternal ABX can produce long-lasting effects on the gut microbiome and neuroimmune responses of offspring. These findings support the role of the early microbiome in the development of offspring gastrointestinal and immune systems.

## 1. Introduction

Autism spectrum disorder (ASD) comprises a group of heterogeneous neurodevelopmental disorders best characterized behaviorally by their impairments in communication, social interactions, and repetitive and/or restrictive interests and behaviors. There are physiological impairments that often precede and/or exacerbate the behavioral symptoms. These impairments are not strictly neuronal in nature; we and others have shown that there is frequently an increase in inflammatory cytokines, immune cell responses, and altered microbiota profiles, often associated with worsening behavior [1,2,3,4,5,6,7,8]. Gastrointestinal (GI) symptoms have commonly been reported in individuals with ASD, with over half experiencing some type of GI comorbidity [9]. Despite this, there remain gaps in our understanding of underlying physiological mechanisms connecting GI symptoms with ASD impairments. The gut microbiota comprises a variety of microorganisms that live in the GI tract and play essential roles in health and disease [10]. They can ferment non-digestible substrates that support the growth of subpopulations of microbes, including those that produce short-chain fatty acids (SCFAs), such as butyrate [11]. Dysbiosis (or altered composition) can result in blooming (overgrowth) of under-represented and/or potentially harmful microbes [12], with or without diminishing otherwise health-promoting microbe populations. In addition, SCFAs and other bacterial metabolites have been shown to have a bidirectional influence on the central nervous system (CNS) with implications for neuronal development and behavior. This bidirectional communication between the GI and CNS is the microbiota–gut–brain (MGB) axis [13,14,15].

As part of the MGB axis, microbes can trigger the release of neurotransmitters by signaling via toll-like receptors (TLR) and other pattern recognition molecules on epithelial, immune, and neuronal cells, affecting physiological processes throughout the CNS [16,17,18]. In addition, bacteria can alter the metabolism of neurotransmitters; for example, *Clostridium* can induce decarboxylation of tryptophan to tryptamine affecting serotonin production [19], while *Lactobacillus* can metabolize glutamate into the neuronal inhibitor GABA [20]. There are multiple routes for which molecules involved in the MBG axis (i.e., neurotransmitters, microbial metabolites, and secreted cytokines) can pass the gut vascular barrier (GVB) and ultimately reach the brain after crossing the blood–brain barrier (BBB) [21,22,23]. The MGB axis has been proposed to be involved in disorders involving neuroimmune interactions, including ASD. These disorders most often present with sex bias, suggesting the involvement of sex differences in immune responses and gut-microbiota homeostasis [24,25,26]. The microbiota has also been shown to participate in the maturation of the immune system; this occurs during postnatal gut colonization and is thought to persist into adulthood, making them potential modulators of immune responses throughout the lifespan [27,28]. An invaluable tool that provides insight into the MGB axis is antibiotic-induced depletion/dysbiosis of the microbiota. Dysbiosis of the gut microbiota, as well as inflammation, play key roles in the pathogenesis of several immunological diseases not limited to ASD [29,30].

Studies of environmental risk factors of ASD have included maternal infections/inflammation or antibiotics during the prenatal and the early postnatal period [31,32]. Prenatal maternal immune activation (MIA) has been used to model altered neurodevelopment in animals, including rodents and non-human primates. It may help elucidate potential mechanisms in disorders such as ASD or schizophrenia. In addition to recapitulating some of the behavior defects seen in neurodevelopmental disorders [33,34], these animal models have demonstrated that MIA leads to microbiota dysbiosis as well as impaired GVB function [35,36], and alterations in the immune profiles of offspring [37,38]. Several epidemiological studies also have shown an association between antibiotic (ABX) use during pregnancy and increased risk of developing ASD [39,40,41], potentially due to altering the microbiome. Together these studies demonstrated the influence that the early microbiome and ABX have on offspring brain and, subsequently, behavior. Conversely, in MIA offspring, ABX administered over time to eradicate the gut microbiota dampened the degree of the maternal immune response during pregnancy, and subsequent effects on behavior and immune responses in the offspring were blunted [42,43,44,45,46].

The limitations of epidemiological studies on antibiotic administration during gestation can be overcome by using animal models which offer unique utilities, such as longitudinal evaluation from birth through adulthood. ABX models could represent a valuable tool, giving insight into how maternal administration of ABX may contribute to changes in neuroimmune responses and offspring phenotype. Thus, in a modification to the Rakoff-Nahoum et al. protocol, we generated a maternal ABX model by giving an ABX-cocktail consisting of vancomycin 0.5 mg/mL, ampicillin 1 mg/mL, and neomycin 1 mg/mL, a treatment that is known to cause severe dysbiosis [17], during pregnancy. ABX were removed immediately following birth for the maternal microbiome to recover and foster normal colonization of bacteria from environmental sources, which, together with diet, may influence the overall composition of the gut microbiota. This allows us to precisely pinpoint the effects of maternal ABX during gestation and parturition. Removal of antibiotics at birth was also intended to minimize the direct impact on the offspring’s gut microbiota during nursing, as antibiotics administered to the mother can reach breastmilk [47]. Breastmilk, free of ABX, is associated with basic essential nutrients, passive antibody transfer, and microbiota that have considerable health benefits for the offspring [48]. Additionally, it has been demonstrated that the most consequential timing of perinatal ABX was when giving in drinking water to dams and withdrawn at birth, rather than directly to pups or indirectly through breastmilk to pups after birth [49,50].

As components of the ABX-cocktail can be absorbed through the GI tract, we included a vancomycin alone group as an additional control as it does not cross the GVB when administered orally, and targets only Gram-positive bacteria [13]. To adequately assess the immune profile of offspring, responses to the potent immunogen LPS were evaluated in offspring from ABX treated dams and vehicle control. To assess the stability of the model, our mice were weaned at postnatal day 21 (p21); these served as the prepubescent (juvenile) mice in which secondary sex characteristics start to develop and 6-month mice served as mature adults in this study.

Gut microbiota influence motor activity and anxiety-like behaviors; these studies suggest that gut microbiota can also affect the development of the brain and eventually alter behavior [51,52,53,54]. Our study assesses how antibiotic use during pregnancy alters offspring outcomes by assessing microbiome and immune profiles that predate behavioral defects and determines if these alterations persist into adulthood. Significant sex differences occur in neurodevelopmental disorders and in animal models of neurodevelopment, and these are addressed here. Results from this study highlight that ABX administered during the late prenatal period through birth have long-lasting effects on the immune system of offspring, including neuroimmune responses and changes in the composition of the microbiome. These effects could have long-term consequences for neurodevelopment and subsequent behavior.

## 2. Materials and Methods

### 2.1. Experimental Animals

All breeders are C57BL/6J from Jackson Laboratory (Sacramento, CA, USA). The mice were allowed to acclimate to our vivarium before any breeding. This study utilized both male and female offspring. The ages were selected to represent development time points in a mouse, i.e., from postnatal day 21 (p21) juvenile to 6 months (6m) mature adult mouse. Mice were fed the same standard chow throughout the study and were housed under specific pathogen-free conditions with a 12/12 h light/dark cycle at the UC Davis Medical Center campus. This study was performed in consideration of alleviation of pain and suffering. The Institutional Animal Care and Use Committee at the University of California Davis approved these animal studies.

### 2.2. Maternal ABX

Mice were bred between 6 and 8 weeks of age. Trio breeding was used to increase breeding efficiency, three cages per group totaling six dams. The embryonic (E) day was determined by the presence of a vaginal plug marking E0.5, the males were then removed from the cage. Dams were either untreated (naive group) or treated orally via drinking water from E14.5 until parturition, i.e., 5–7 days with either vancomycin (0.5 mg/mL) (Alvogen, Morristown, NJ, USA) alone (vancomycin group) or ampicillin (1 mg/mL), neomycin (1 mg/mL) (MilliporeSigma, Burlington, MA, USA) and vancomycin (0.5 mg/mL) in tandem (ABX-cocktail group). ABX treatment did not affect water consumption or chow. Fresh antibiotics were maintained in the drinking water until birth, where they were then switched back to regular drinking water. After birth, dams were placed in individual cages. The average litter size for these first-time breeders was 7–8 pups and ABX did not alter litter size. This study focused on the effects of maternal ABX during pregnancy on the offspring, so they were kept in their groups 2–4 per cage. The untreated naive group served as the control. Each experimental time point consisted of 1 male and 1 female from each of the groups of dams.

### 2.3. Systemic Immune Challenge

We induced a robust systemic immune response by intraperitoneal (IP) injection with 5 mg/kg of body weight LPS from *Escherichia coli* O55:B5 (MilliporeSigma, Burlington, MA, USA) into offspring mice at p21 and 6m. Equivalent volumes of PBS were administered as a control. Mice were euthanized for tissue and fecal pellets collection 24 h post-injections. This allowed for enough time for signal transduction, transcription, and protein synthesis to produce a peak CNS immune response. It also provided time for gut microbiota changes.

### 2.4. Sample Collection

Mice were placed individually in sterile cages to collect fecal pellets, without risk of contamination. The collected samples were stored directly on dry ice before being transferred to −80 °C until analysis. Animals were then anesthetized with isoflurane. Blood was drawn into an uncoated capillary tube via a nick in the cardiac artery. The blood was transferred to a tube and allowed to clot at room temperature before placing it on ice. After a 10 min centrifugation at 4 °C and 12,000 rpm, blood serum was collected and stored at −80 °C until analysis. Following blood collection, brains were perfused with HBSS through the left ventricle, then the extracted brain was dissected. The extracted brain was hemi-sectioned, and the hippocampus was exposed by peeling away the cortex layer; the exposed hippocampal horn was then also excised. The cecum was then removed from the gut. All tissue samples were immediately frozen on dry ice and then stored at −80 °C until analysis.

### 2.5. Microbiota Analysis

Bacterial DNA was extracted from the frozen fecal samples of the naive, vancomycin, and ABX-cocktail groups at p21 and 6m. Using barcoded primers, the 16S rRNA amplicon sequencing of the V4 region was performed using the MiSeq platform (Illumina, San Diego, CA, USA). In brief, the Illumina demultiplexing, merging of the reads and trimming of the barcodes and primers, and the QC were performed by the UC Davis sequencing core. FastQ files were then analyzed using the QIIME2 software package v. 2021.2 [55]. We first used the DADA2 plugin [56], which was used to filter and trim the files reads, find alternative sequence variants (ACVs), remove chimeras, and then count abundance. We then used the q2-feature-classifier plugin [57], which was used to identify the most likely original taxonomic sequences in the sample based on the SILVA 138 database [58] and can be represented on bar plots. Finally, we used the q2-emperor plugin, which was used to view sample taxonomic diversity profiles. β-diversity was represented on PCoA plots.

### 2.6. Cytokine Measurement

Serum was collected from the clotted blood samples. Brain samples were collected from mice and immediately stored at −80 °C until use. Individual brain samples were suspended in PBS with a protease inhibitor cocktail (Cell Signaling Technology, Danvers, MA, USA), then physically disrupted by vortexing thoroughly followed by brief sonication. The disrupted pellets were centrifuged (30,000× *g* for 10 min at 4 °C) to precipitate insoluble materials. The hippocampus was processed similarly to the whole brain. The concentration of protein extracts in the supernatant of the brain and hippocampus were measured by using the BCA protein assay (Thermo Scientific, Waltham, MA, USA). The quantification of cytokines in samples was then determined using mouse reactive Milliplex™ multiplexing bead immunoassays (Millipore, Burlington, MA, USA). Serum was run at equal volumes, while brain supernatant was run at 3.2 μg/μL in accordance with the instructions of the manufacturer’s protocol. In brief, the supernatant was incubated with antibody-coupled beads. After a series of washes, a biotinylated detection antibody was added to the beads, and the reaction mixture was detected by the addition of streptavidin-conjugated to phycoerythrin. The bead sets were analyzed using a flow-based Luminex™ 100 suspension array system on the Bio-Plex 200 platform (Bio-Rad Laboratories, Hercules, CA, USA). The unknown sample cytokine concentrations were then calculated by Bio-Plex Manager software using a standard curve derived from the known reference cytokine concentrations supplied by the manufacturer.

### 2.7. Metagenome Computation Analysis

16S ASV datasets were used to predict metagenome functional content in each sample of the naive, vancomycin, and ABX-cocktail groups. Briefly, the 16S ASVs were normalized and aligned to a reference phylogenetic tree then the predicted functional gene families and copy numbers for each specific ASV were found. We ran the full PICRUSt2 pipeline command in QIIME2. As output, we obtained Kyoto Encyclopedia of Genes and Genomes (KEGG) Orthology metagenome predictions [59] and MetaCyc pathway abundance predictions based on enzyme profile enzyme commission (EC) code and abundances [60]. Linear discriminant analysis (LDA) effect size (LEfSe) is an algorithm that identifies genomic features characterizing the differences between biological groups. We used this to compare the naive, vancomycin, and ABX-cocktail groups. We used the LPS treatment as a subclass in this analysis. The grouped data were analyzed using the non-parametric factorial Kruskal–Wallis with a significance set to <0.05, and pairwise Wilcoxon’s tests. Finally, LEfSe uses LDA to estimate the effect size of each differentially abundant feature [61]. The LDA threshold was set at ±2. LEfSe data analysis was prepared using the Huttenhower Lab Galaxy server.

### 2.8. Statistical Analysis

Statistical differences in abundances of gut microbiota were analyzed using a two-way ANOVA followed by the Tukey post-hoc test for multiple comparisons. Data are presented as mean ± standard error of the mean. Gut microbiota principal coordinate analysis (PCoA) was performed using Emperor. Stack bar plots were generated in RStudio v 1.4.1103 software (RStudio, Boston, MA, USA). The Kruskal–Wallis test was used for the analysis of cytokine data followed by Dunn’s multiple comparison testing. Significant differences between the three antibiotic groups were represented by *, with * *p* < 0.05, ** *p* < 0.01, *** *p* < 0.001, and **** *p* < 0.0001. Nonsignificant differences are not indicated in the figures. All the analyses were performed using Qiime2 software and GraphPad Prism v 9.0 Software (GraphPad, La Jolla, CA, USA). Metagenome analysis used Kruskal–Wallis with a significance set to *p* < 0.05, and pairwise Wilcoxon’s tests. LEfSe analysis revealed significant bacterial differences in gut microbiota between the ABX-treated groups and the naive group. LDA scores (log10) > 2 and *p* < 0.05 are shown. Final figures were assembled using Biorender.com v 2022 (Toronto, ON, Canada).

## 3. Results

### 3.1. Maternal ABX Decreases the Diversity of Offspring Gut Microbiota

**Figure 1:**
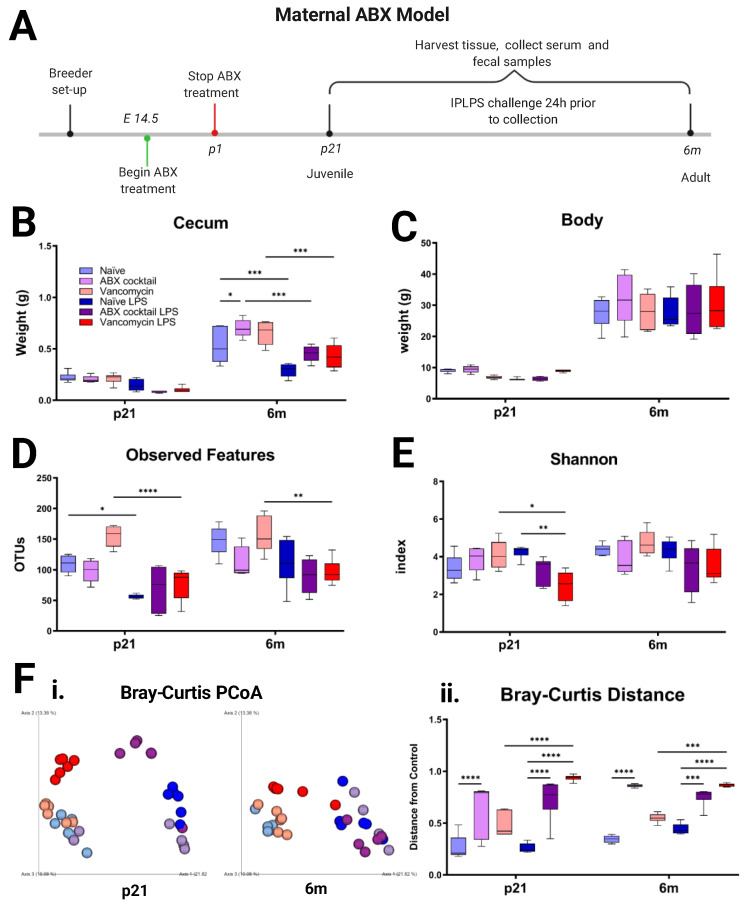
Prenatal ABX administration alters the gut’s microbiota diversity. (**A**) Experimental timeline showing maternal ABX treatment, offspring LPS challenge, and collection days. (**B**,**C**) Effect of ABX treatment and LPS challenge on cecum weight and body weight. (**D**,**E**) α-diversity indexes using OTUs and Shannon index based on the Illumina sequencing data (**F**) β-diversity indexes using (i) principal coordinate analysis (PCoA) plot and (ii) box plots of distances based on Bray–Curtis dissimilarity between samples. Each color represents a unique prenatal exposure baseline combined with or without stimulation: naive = light blue, vancomycin = light red, ABX-cocktail = light purple, naive LPS = blue, vancomycin LPS = red, and ABX-cocktail LPS = purple. Naive, vancomycin, and ABX-cocktail samples were run in parallel, the number of mice in each group were male *n* = 3 and female *n* = 3. The levels of significance (*p*-values) were determined by two-way ANOVA test followed by Tukey post-correction test for multiple comparisons. The values represent means + SEM, * *p* ≤ 0.05, ** *p* ≤ 0.01, *** *p* ≤ 0.001, and **** *p* ≤ 0.0001.

To assess the impact of maternal ABX on offspring gut microbiota and immunity, pregnant dams were orally administered ABX on embryonic day 14.5 (E14.5) until the birth of their pups. The offspring were analyzed at p21 and 6m (Figure 1A). We measured body and cecum weights and found no significant differences in body weight across groups or after in vivo LPS challenge (Figure 1B). The ABX-cocktail adults had significantly larger cecum (*p* ≤ 0.05), while the cecum weights were significantly decreased (*p* ≤ 0.001) following LPS challenge in all groups (Figure 1C and Figure S1). Changes in cecum size have been associated with altered gut composition and or GI motility. We then analyzed the gut microbiota by Illumina sequencing of the 16S rRNA gene of bacterial DNA extracted from fecal samples. We determined the diversity and differential abundance of operational taxonomic units (OTUs). Diversity can be measured as the degree of heterogeneity within a group or the difference between two groups. The α-diversity indexes are used to determine the ecological diversity within microbial communities [62].

We found that gut microbiota α-diversity was not significantly altered by the ABX-cocktail treatment (Figure 1D,E). Though none reached significance at baseline, there were decreases in species richness in the offspring of the ABX-cocktail group as demonstrated by lower observed OTUs at p21 and at 6m (Figure 1D). β-diversity indexes involve distance metrics used for differences between microbial communities. There were significant differences observed in β-diversity in the ABX-cocktail group (Figure 1F). Principal coordinate analysis (PCoA) comparing Bray–Curtis showed the ABX-cocktail group cluster separately from the naive group, and this distance was significant (*p* ≤ 0.0001) at both p21 and 6m (Figure 1F), with or without LPS stimulation. Generally, gut microbiota diversity and richness were reduced in the cocktail group in both sexes, depending on age.

In the vancomycin alone treated group, the same concentration of vancomycin (namely, 0.5 mg/mL) as in the ABX-cocktail group was used. However, in contrast, the vancomycin group showed a trend toward an increase in observed OTU and Shannon index decreases at p21 (*p* ≤ 0.01) (Figure 1C, D). However, the vancomycin group microbiota displayed significantly altered α-diversity without a distinct community clustering or a significant distance from controls. Though these shifts from naive offspring mice also did not reach significance, we saw that the cocktail and vancomycin groups are significantly different from each other. The vancomycin group also clustered close with the naive group in Bray–Curtis as demonstrated by the overlap (Figure 1F) and had no significant differences in distance. However, following LPS stimulation, vancomycin groups were significantly (*p* ≤ 0.0001) separated from the controls. Though some studies have shown that early life vancomycin treatment decreased diversity in offspring, they often allowed for postnatal treatment of dams beyond the first few days of life [63,64,65,66,67,68,69].

LPS challenge in vivo can affect α- and β-diversity [70,71]. In our model, we found that LPS challenge decreased α-diversity in all groups, significantly in the naive and vancomycin groups at p21 (*p* ≤ 0.05) and (*p* ≤ 0.0001), but not 6m (Figure 1C–E). LPS-treatment also significantly decreased the Shannon index in the vancomycin group at p21 (*p* ≤ 0.05). The β-diversity shift was less noticeable in the ABX-cocktail group due to their already clustering separately from the unstimulated naive group. In general, LPS stimulation leads to an alteration of the dysbiotic profile as measured by these diversity indexes.

### 3.2. Offspring Gut Microbiota Homeostasis Is Altered by Maternal ABX

**Figure 2:**
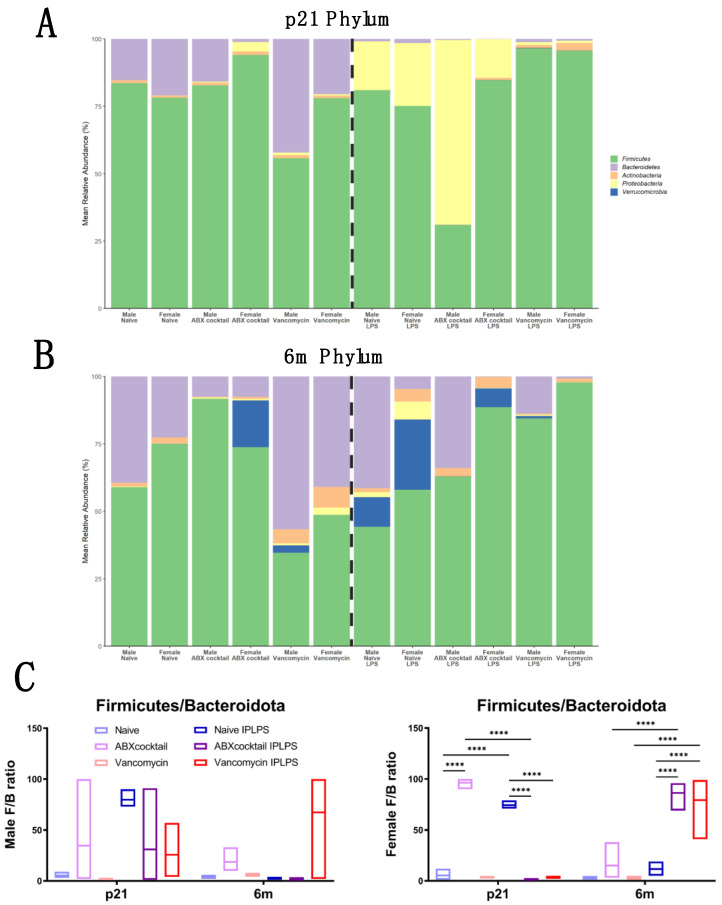
Prenatal ABX administration alters offspring gut microbiota taxonomy. (**A**,**B**) Relative abundances of sequences classified to phylum at p21 and 6m, respectively, with *Firmicutes* being the most abundant in both. Only phyla with a significant number of reads are shown. (**C**) *Firmicute*s/*Bacteroidetes* ratios where bars represent the group mean. Each color represents a unique prenatal basal exposure combined with or without LPS stimulation: naive = light blue, vancomycin = light red, ABX-cocktail = light purple, naive LPS = blue, vancomycin LPS = red, and ABX-cocktail LPS = purple. Naive, ABX-cocktail, and vancomycin samples were run in parallel, the number of mice in each group were male *n* = 3 and female *n* = 3. The levels of significance (*p*-values) determined by two-way ANOVA test followed by Tukey post-correction test for multiple comparisons. The bar values represent means + SEM, **** *p* ≤ 0.0001.

Given the response the maternal ABX-cocktail had on the gut microbiota diversity of offspring, we next examined how this altered diversity contributes to different taxonomic profiles. There was a total of 72 fecal samples obtained for sequencing from the naive, ABX-cocktail, and vancomycin groups. The relative abundance of the gut microbiota composition was first compared for bacterial phyla present in the samples. In all of our C57BL/6J naive mice, the most dominant bacteria phyla belonged to *Firmicutes* and *Bacteroidetes*, accounting for more than 98% of the assigned sequences. The remaining phyla belonged to *Proteobacteria*, *Actinobacteria*, and *Verrucomicrobia*, with relative abundances that were relatively low or isolated (Figure 2A,B, Table S1). When naive males and females from p21 were examined, variations at the phylum level were evident, where the *Firmicutes* were 84% and 78%, the *Bacteroidetes* were 15% and 21%, and lastly, the phylum *Actinobacteria* accounted for 1% in both male and female naive controls. We next examined the ABX-cocktail group to see how maternal ABX shifted populations at the phyla level. They did not alter the p21 male phyla composition substantially with *Firmicutes* at 83% and *Bacteroidetes* were 16%. However, females in the ABX-cocktail group experienced a loss of *Bacteroidetes*, with *Firmicutes* increasing to 94%, and *Proteobacteria* now 4%, *Actinobacteria* and *Bacteroidetes* both accounted for the remaining 1%. In contrast, the p21 vancomycin males had decreased *Firmicutes* at 56% and increased *Bacteroidetes* at 42%, while the p21 females were similar to naive controls, with *Firmicutes* at 78% and *Bacteroidetes* at 21% (Figure 2A, Table S1). In the 6m male and female naive controls, the *Firmicutes* were 59% and 75%, and *Bacteroidetes* were 39% and 23% respectively, with *Actinobacteria* accounting for the remaining populations. When we examined the ABX-cocktail group at the phyla level, the 6m males and females also had differences in populations. The 6m male phyla composition was *Firmicutes* at 92% and *Bacteroidetes* were 8%. However, females in the ABX-cocktail group experienced a loss of *Firmicutes* 74% similar to the naive controls, with *Bacteroidetes* 8% similar to the males, with *Verrucomicrobia* now accounting for 17%, and *Actinobacteria* accounting for the remaining 1%. The 6m vancomycin males had decreased *Firmicutes* at 35% and increased *Bacteroidetes* were 57%, while the 6m vancomycin female *Firmicutes* were 49% and *Bacteroidetes* were 41%. The remaining phyla from both sexes came from *Actinobacteria, Proteobacteria*, and *Verrucomicrobia* (Figure 2B, Table S1). Variations at the phylum level were evident at baseline in both the p21 and 6m.

Following in vivo LPS challenge phyla changes have been detected [70,72]. We found that LPS challenge had a significant effect on the microbiota at both p21 and 6m timepoints, depending on the maternal ABX treatment or offspring sex. For instance, in the p21 naive LPS males and females, *Firmicutes* were 81% and 75%, and *Proteobacteria* were 18% and 23%, respectively, with *Bacteroidetes* accounting for the remaining populations. At 6m, LPS challenge also altered phyla in naive male and females, *Firmicutes* were 44% and 58%, and *Bacteroidetes* were 41% and 5%, respectively.

When we examined the ABX-cocktail group following LPS to see how maternal ABX shifted populations at the phyla level, the p21 male phyla composition changed substantially with *Firmicutes* decreasing to 31%, *Bacteroidetes* increasing to 34%, and *Proteobacteria* increasing to 69%. However, p21 females in the ABX-cocktail group experienced a loss of the phylum *Bacteroidetes*, with *Firmicutes* increasing to 85%, and *Proteobacteria* increasing to 14%; 6m ABX males with *Firmicutes* increased to 63%, and *Bacteroidetes* to 34%; 6m females in the ABX-cocktail group experienced a loss of the phylum *Bacteroidetes*, with *Firmicutes* increasing to 89%, and *Actinobacteria* accounting for the remaining population.

After LPS, the vancomycin group had predominantly *Firmicutes* as the remaining phylum, with p21 male at 97%, p21 females at 96%, 6m males at 85%, and 6m females at 98%. Following LPS, the 6m males also sustained some *Bacteroidetes* at 14% while p21 males and females of both ages had decreased *Bacteroidetes* to less than 1% (Figure 2A,B). LPS challenge decreased *Bacteroidetes* in both sexes at p21 and 6m, which affects their relationship to *Firmicutes*. The comparison of the relative abundance of these two phyla is described as the *Firmicutes/Bacteroidetes* (F/B) ratio. An increased F/B ratio leads to less favorable outcomes and has been associated with dysbiosis and metabolic disorders. When comparing the F/B ratio of our samples, there was a significant increase in the F/B ratio in the p21 ABX-cocktail females (*p* ≤ 0.0001) as compared to the naive control; however, the males were not significant. The 6m males and females had increased F/B but they did not reach significance following correction for multiple comparisons. The vancomycin group had F/B ratios similar to the naive controls without LPS stimulation. However, after LPS stimulation, female offspring from both treatment groups showed alterations in the F/B ratio (*p* ≤ 0.0001), but males did not reach significance at any age (Figure 2C).

### 3.3. Gut Microbiota Relative Abundance Is Altered in a Sex-Dependent Manner

**Figure 3:**
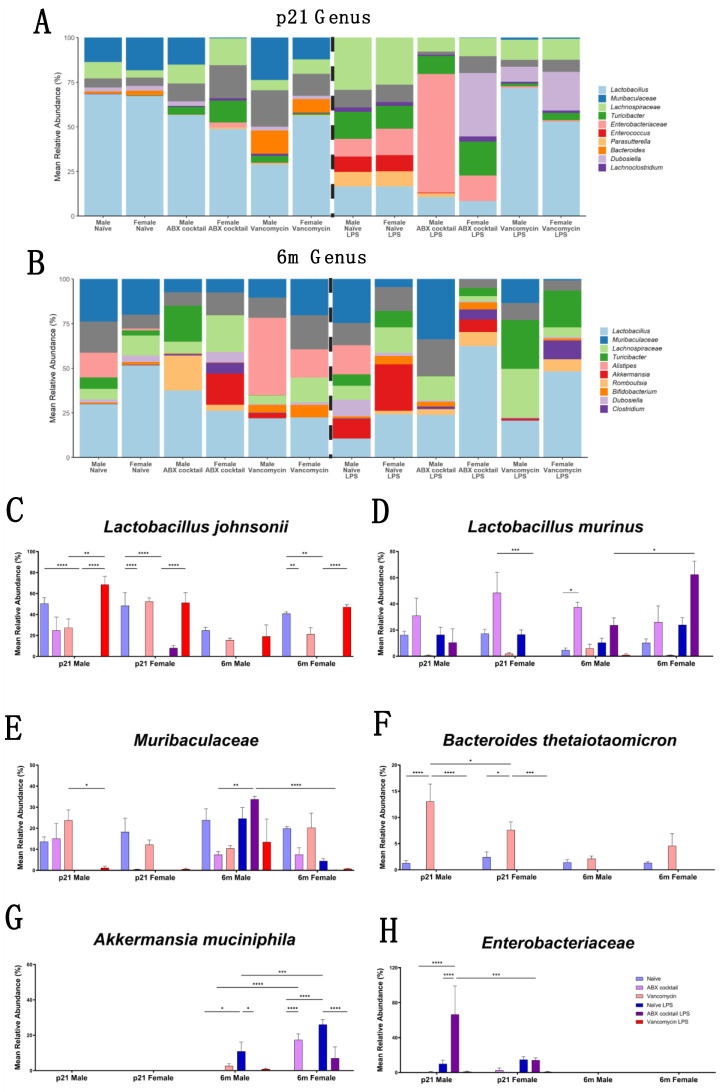
Taxonomy representation at genus and species level. (**A**,**B**) Relative abundances of sequences classified to genus at p21 and 6m respectively, with *Lactobacillus* being the most abundant in both. Only the top 10 genera in each are shown, the remaining are summed and greyed. (**C**) *Lactobacillus johnsonii*, (**D**) *Lactobacillus murinus*, (**E**) *Muribaculaceae*, (**F**) *Bacteroides thetaiotaomicron*, (**G**) *Akkermansia muciniphila*, and (**H**) *Enterobacteriaceae*. Each color represents a unique prenatal basal exposure combined with or without stimulation: naive = light blue, vancomycin = light red, ABX-cocktail = light purple, naive LPS = blue, vancomycin LPS = red, and ABX-cocktail LPS = purple. Naive, vancomycin, and ABX-cocktail samples were run in parallel, the number of mice in each group were male *n* = 6 and female *n* = 6. The levels of significance (*p*-values) determined by two-way ANOVA test followed by Tukey post-correction test for multiple comparisons. The bar values represent means + SEM, * *p* ≤ 0.05, ** *p* ≤ 0.01, *** *p* ≤ 0.001, and **** *p* ≤ 0.0001.

We further characterized the impact of maternal ABX treatment on the offspring taxonomy by comparing the relative abundance of the gut microbiota composition at the genus levels using 16S rRNA data [73]. The genus *Lactobacillus* were the most abundant bacteria in our naive controls at both the p21 and 6m timepoints (Figure 3A,B). *Lactobacillus johnsonii* contributed an average of greater than 40% of the total relative abundance. In the ABX-cocktail group, *Lactobacillus johnsonii* was decreased in the males at p21 and 6m, but this did not reach statistical significance after correction for multiple comparisons even though 6m males had complete depletion; however, the females had a significant decrease that depleted this bacteria entirely at p21 (*p* ≤ 0.0001) and 6m (*p* ≤ 0.01). Vancomycin alone did not significantly alter *Lactobacillus johnsonii* in the offspring in either sex at p21 or 6m. LPS resulted in robust decreases of *Lactobacillus johnsonii* in naive p21 mice (*p* ≤ 0.0001) of both sexes. A significant reduction was also seen at 6m. This same pattern was seen in ABX-cocktail male mice at p21 only, as 6m males and all females had negligible amounts of *Lactobacillus johnsonii* prior to LPS stimulation. The vancomycin mice did not experience a decrease in *Lactobacillus johnsonii* following LPS, rather they were significantly increased (*p* ≤ 0.0001) as compared to naive controls, except for 6m males where increases did not quite reach significance. Surprisingly, there was an increase in *Lactobacillus johnsonii* after LPS for p21 males (*p* ≤ 0.01) (Figure 3C). *Lactobacillus murinus* was the next most abundant in the gut microbiota in control mice, accounting for an average of 20% at p21 and 15% at 6m. ABX-cocktail mice had increased *Lactobacillus murinus* as compared to naive controls. However, this was only significantly increased (*p* ≤ 0.05) in the 6m males after correction for multiple comparisons. Vancomycin mice had drastically depleted *Lactobacillus murinus* in all groups except 6m males that had levels comparable to the controls. LPS treatment did not result in significant changes in the abundance of *Lactobacillus murinus* in naive or ABX-cocktail males at either timepoint; however, *Lactobacillus murinus* decreased (*p* ≤ 0.001) at p21 and increased (*p* ≤ 0.05) in 6m female ABX-cocktail mice treated with LPS as compared to males. No differences in these bacteria were seen in any of the vancomycin mice after LPS (Figure 3D).

*Muribaculaceae* were the most abundant family from the phyla *Bacteroidetes*. These uncultured bacteria species from the *Muribaculaceae* family accounted for approximately 15–25% of the total relative abundance of the gut microbiota of our naive controls, depending on sex and age. In the ABX-cocktail group *Muribaculaceae* was depleted in female p21 mice and was not restored at the 6m time point. *Muribaculaceae* in p21 ABX-cocktail males were unaffected; however, at 6m, abundance was reduced. LPS resulted in robust decreases of *Muribaculaceae* in all groups except in 6m males. Surprisingly, after LPS challenge the 6m ABX-cocktail males had significantly increased *Muribaculaceae* (*p* ≤ 0.01) (Figure 3D). Another member of the phylum *Bacteroidetes* is *Bacteroides thetaiotaomicron* which was present in all the naive mice. The ABX-cocktail group did not have *Bacteroides thetaiotaomicron*; however, the vancomycin had significantly more than the naive control p21 males (*p* ≤ 0.0001) and p21 females (*p* ≤ 0.05). *Bacteroides thetaiotaomicron* was depleted entirely from the naive and vancomycin groups following LPS challenge, despite vancomycin p21 having a significantly higher abundance than the naive mice, while the ABX-cocktail did not have any before or after LPS challenge (Figure 3E).

In addition to diminishing common commensal microbe populations, in vivo LPS challenge has the propensity to alter the gut microbiota such that the gut could be a more favorable environment for the growth of under-represented and/or potentially opportunistic microbes [74,75,76]. We saw increases in *Akkermansia muciniphila* (*Verrucomicrobia*) and *Enterobacteriaceae* (*Proteobacteria*) in response to LPS challenge, depending on sex and treatment group (Figure 3F,G). The unclassified genera in family *Enterobacteriaceae* had the most significant change in the p21 male ABX-cocktail group (*p* ≤ 0.0001) compared to naive controls, with substantially increased *Enterobacteriaceae* (*p* ≤ 0.0001) after LPS treatment. Female p21 mice also had an expansion of *Enterosbacteriaceae* after LPS, but to a less significant degree than males (*p* ≤ 0.001). The increases in *Enterobacteriaceae* were not seen at 6m. *Akkermansia muciniphila* increased in males (*p* ≤ 0.05) and females (*p* ≤ 0.0001) of the naive group at 6m after LPS, with higher increases in females (*p* ≤ 0.001). The 6m female ABX-cocktail group had increased *Akkermansia muciniphila* (*p* ≤ 0.0001) comparing naive controls; however, after LPS stimulation, there is no significant difference. *Bifidobacterium pseudolongum* belonging to the phyla *Actinobacteria*, along with additional phyla and genera, had significant alterations in response to LPS that also varied in a sex-dependent manner, which was greatly influenced by age (Figure S1). Overall, ABX treatment led to an alteration in gut microbiota ranging from significant increases to entire depletion of whole populations, while others had no significant differences regardless of sex.

### 3.4. Maternal Gut ABX Influences CNS Cytokine Levels in the Offspring

**Figure 4:**
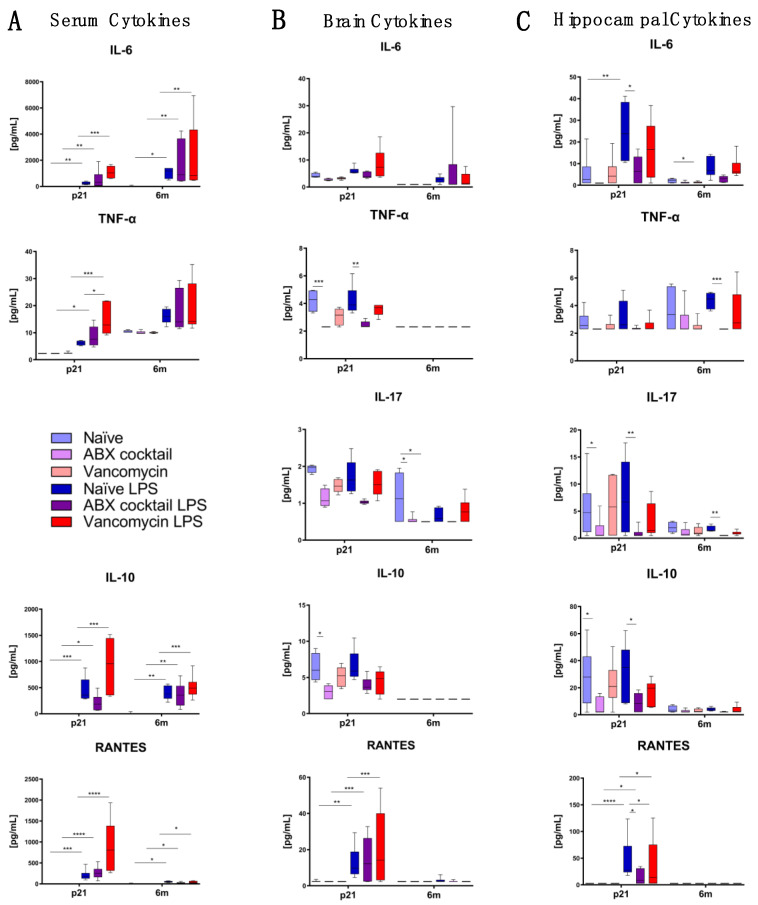
Inducible cytokines are affected by ABX exposure. Luminex data showing the responses of IL-6, TNFα, IL-17, IL-10, and RANTES from (**A**) serum (**B**) brain and (**C**) hippocampus. Each color represents a unique prenatal basal exposure with or without LPS stimulation: naive = light blue, vancomycin = light red, ABX-cocktail = light purple, naive LPS = blue, vancomycin LPS = red, and ABX-cocktail LPS = purple. Naive, vancomycin, and ABX-cocktail samples were run in parallel. The levels of significance (*p*-values) determined by Kruskal–Wallis with Dunn’s multiple comparison test. Values represent median (min-max), * *p* ≤ 0.05, ** *p* ≤ 0.01, *** *p* ≤ 0.001, and **** *p* ≤ 0.0001.

We next assessed whether altered gut microbiota in the offspring resulting from maternal ABX treatment influenced cytokine patterns in the periphery and brain. Luminex multiplex cytokine assay was performed, starting with serum samples in an effort to characterize peripheral inflammation. Serum cytokines in the treatment groups were generally not different from the naive controls at either timepoint, except for reduced production of IFN-γ (*p* ≤ 0.05) by ABX-cocktail mice compared to the naive controls at 6m (Figure S3). These results show that although ABX-cocktail resulted in altered gut microbiota, this generally did not significantly alter the serum concentration of pro-inflammatory cytokines. However, following LPS challenge, cytokines increased in each group, as expected. However, cytokines increased in the ABX-cocktail offspring significantly more than the naive controls after LPS challenge (Figure 4A and Figure S2).

We next characterized cytokines in the CNS by measuring whole brain cytokine levels. The ABX-cocktail group produced significantly less TNF-α is (*p* ≤ 0.0001) and IL-10 (*p* ≤ 0.05) at p21, and IL-17 (*p* ≤ 0.05) at 6m (Figure 4B). Due to the role of the hippocampus in social memory and behavior [77] and the fact that it is highly vulnerable to external stimuli we next measured cytokines in this specific region. Here, the ABX-cocktail group produced significantly less IL-10 (*p* ≤ 0.05) and IL-17 (*p* ≤ 0.05) at p21 (Figure 4C), and significantly less IFN-γ (*p* ≤ 0.05) at 6m (Figure S3). The vancomycin group cytokines were not significantly different from the naive controls, except for decreased IL-17 (*p* ≤ 0.05) in the whole brain and decreased IL-6 (*p* ≤ 0.05) in the hippocampus, both at 6m.

Following LPS challenge, cytokines increased overall in serum and brain tissue at both timepoints; however, this differed depending on the group (Figure 4 and Figure S2). In the ABX-cocktail group, LPS treatment induced significantly less peripheral IL-1α at 6m (Figure S3), significantly less TNF-α (*p* ≤ 0.001) and IL-1α (*p* ≤ 0.05) in the brain at p21 (Figure 4B) compared to the LPS treated naive control. In the hippocampus, LPS-treated ABX-cocktail mice produced significantly less IL-6 (*p* ≤ 0.005), IL-10 (*p* ≤ 0.05), IL-17 (*p* ≤ 0.01), and Regulated on Activation, Normal T cell Expressed and Secreted (RANTES) (*p* ≤ 0.05) at p21 and TNF-α (*p* ≤ 0.001) and IL-17 (*p* ≤ 0.01) at 6m when compared to the control mice (Figure 4C). Overall, the vancomycin group had significantly higher serum cytokines while the ABX-cocktail had significantly less cytokines in the CNS. Therefore, we confirmed that maternal ABX alters offspring neuroimmune responses.

### 3.5. Maternal ABX Alters Predicted Metabolic Pathways

**Figure 5:**
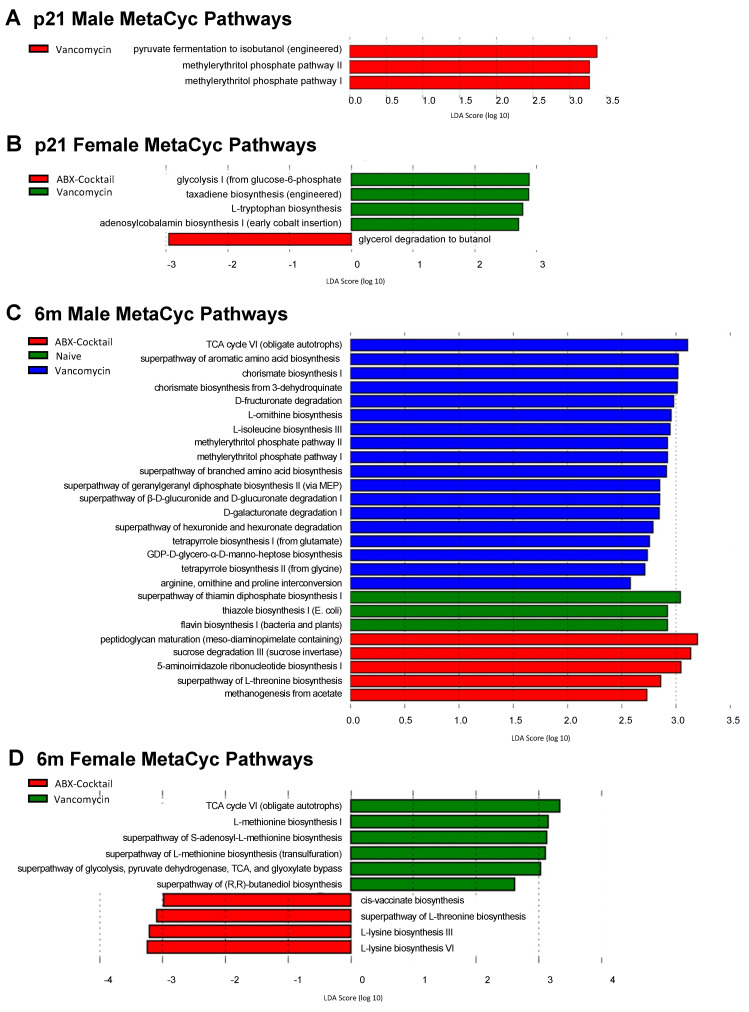
PICRUSt predictions of metabolic pathways. Functional summary for MetaCyc pathways, Linear discriminant analysis (LDA) effect size (LEfSe) analysis revealed significant bacterial differences in gut microbiota between the antibiotic-treated groups and naive group from (**A**) p21 male, (**B**) p21 female, (**C**) 6m male, and (**D**) 6m female. LDA scores (log10) > 2 and *p* < 0.05 are shown. When all three groups are present: naive = green, vancomycin = blue, and ABX-cocktail = red. Otherwise, they correspond to their specific legend. The male *n* = 6 and female *n* = 6 for each group.

To assess the impact of maternal ABX on functional metabolic profiles of offspring gut microbiota, we performed pathway profiling based on predicted metagenomic functions using the bioinformatic tool PICRUSt2 [78] to predict the relative abundances of functional categories and show the deduced functional capacities ranked by the highest effect size associated with the naive, ABX-cocktail, and vancomycin alone groups. The predictive functional capacity of microbiota from each group were first determined by linear discriminative analysis (LDA) effect size (LEfSe) analysis of MetaCyc pathways. A total of 44 MetaCyc pathways were biologically significant of those identified in all samples. The ABX-cocktail male microbiota had no significant changes of pathways at p21 (Figure 5A), while there was a decrease in a single pathway in p21 female microbiota associated with glycerol degradation to butanol, represented by a negative LDA score (Figure 5B). The microbiota from 6m ABX-cocktail males had predicted increases in 5 pathways associated with amino acid production, represented by a positive LDA score (Figure 5C), while in the 6m females there were decreases in four pathways also associated with amino acid production (Figure 5D). Of these, L-threonine biosynthesis was the only pathway affected in both males and females; however, they had opposing LDA scores with 6m female pathways being negatively affected.

In the vancomycin alone group, the methylerythritol phosphate pathway (MEP) I and MEP II were increased in p21 males. In p21 females, a higher abundance of microbial genes associated with glycolysis I from glucose 6-phosphate and L-tryptophan biosynthesis in the vancomycin treatment group. At adulthood, additional pathways were enriched in the vancomycin group—including glycolysis and TCA cycle. Components of the MEP pathway seen in the p21 male mice are still observed in the 6m males adults, with an additional upregulation of the shikimate pathway for the biosynthesis of tryptophan, phenylalanine, and tyrosine [79] in the 6m male vancomycin group (i.e., chorismate biosynthesis I and chorismate biosynthesis from 3-dehydroquinate). Overall, separate pathways were predicted for males and females at p21 and 6m with few exceptions. We see the methylerythritol phosphate pathway (MEP) I/II in males from both ages, and TCA and L-threonine biosynthesis in 6m from male and females.

Analysis of functional capacity was further characterized using KEGG orthologs [60]. The p21 ABX-cocktail males were the only group with negative LDA score for KEGG orthologs (Figure S5). In contrast, the p21 females had increases in only one KEGG ortholog with no decreases. In the ABX group, 6m males had more significantly increased orthologs than the 6m females had, this increase in KEGG ortholog was more than any other group, for instance, p21 and 6m naive females had only one significantly increased ortholog. Overall, our group-wise comparison of functional metabolic profiles only revealed negative LDA scores in the juvenile males from the ABX-cocktail group. Together these data suggest that multiple MetaCyc pathways and KEGG orthologs were affected, most likely due to shifts in the relative abundance of the microbes and their associated metabolic capacities (Figure S4).

## 4. Discussion

Public policy for the use of antibiotics during labor was enacted in the 1990s to prevent sepsis caused by Group B *Streptococcus* species [80], prior to our burgeoning understanding of the importance of the microbiota. Since then, the use of prophylactic antibiotics given during delivery has increased to greater than 30% of all deliveries in the United States [81]. ABX treatment strongly disrupts and reduces the microbial communities in the gut [63,64,65,66,69]; however, much less is known on how maternal ABX impacts the offspring in ABX-treated dams during gestation and there remain significant gaps in our understanding of the health risks of intrapartum antibiotics to the offspring [82,83]. Antibiotics during labor and delivery are known to decrease the diversity of the microbiota in the offspring, including depletion of important early colonizers such as *Bifidobacteria* [84,85,86,87]; thus, research to identify potential long-term effects and outcomes is critically needed, and our study helps to fill this knowledge gap.

Comparing the relative abundance of specific bacteria across taxonomic levels is essential for determining how maternal ABX affects offspring gut microbiota. Overall, we generally found lower diversity within ABX-cocktail samples, differing microbial communities across groups, and changes in gut microbiota that ranged from large expansions to complete loss of populations. Loss of diversity is a typical finding in dysbiosis and is associated with a number of disease states that have risen in prevalence in Western societies where overuse of antibiotics is common. These include, but are not limited to, metabolic disorders (i.e., obesity, type II diabetes), immune-mediated disorders including autoimmune disorders such as multiple sclerosis and rheumatoid arthritis, and gut disorders and inflammatory bowel disease [88]. Reduced diversity and dysbiosis have been seen previously in ASD, and gut dysfunction is common in this disorder [89]; therefore, more research is needed in this area to determine if dysbiosis is contributing pathogenesis of these disorders.

Early colonizers of the gut microbiota alter the environment and affect future populations; for example, bacteria influence the secretion of mucus and antimicrobial peptides by epithelial cells and can alter pH and oxygen tension [90]. Thus, early life microbiota plays a significant role in the future composition of the community. Although not seen in large numbers in mice, *Actinobacteria* are important early colonizers of the human gut, specifically *Bifidobacterium* which predominate in the presence of human breastmilk oligosaccharides [91]. Our findings that maternal ABX can deplete entire phyla, such as *Bacteroidetes* or *Actinobacteria*, could have implications for human infant gut health.

In general, comparing *Firmicutes* to *Bacteroidetes* is considered to be of significant relevance in human gut microbiota composition and a good indicator of gut homeostasis. The ratio between these two phyla has been termed the F/B ratio and is associated with maintaining homeostasis; changes in the F/B ratio have been associated with dysbiosis, with increased ratios associated with inflammatory disorders and obesity [92]. We found an increased F/B ratio in the ABX-cocktail and vancomycin groups following LPS due to decreases in *Bacteroidetes*, namely from *Bacteroides thetaiotaomicron*; however, this increase was only significant in p21 ABX-cocktail females, highlighting sex differences. Both male and female ABX-cocktail mice completely lost *Bacteroides thetaiotaomicron* at both time points and p21 females had a significant reduction the *Muribaculaceae* family which have been shown to correlate with butyrate production [93]. Butyrate and other SCFA play essential roles in maintaining gut immune homeostasis and have the potential to exert multiple effects not limited to influencing gut motility, GVB permeability, and modulating inflammation [94,95]. Members of this family also play a vital role in modulating energy metabolism in mice due to their specialization in the fermentation of complex polysaccharides [96]. The cecum is the microbial fermentation site for many SCFA [97]. The ABX-cocktail group having a significantly increased cecum size was due to the females, highlighting another sex difference.

In this study, *Lactobacillus* populations are drastically influenced by maternal ABX. We can postulate that these dramatic shifts in *Lactobacillus* abundance might alter expression of neuromodulators important for development behavior in the ABX-cocktail group. For example, *Lactobacillus johnsonii* has been shown to increase BDNF in the hippocampus while suppressing TNF-α expression. *Lactobacillus johnsonii* has also been shown to ameliorate some autistic features in mice such as altered cytokines and neuronal signaling pathway [98,99,100]. Other *Lactobacillus* strains, including *L. rhamnosus* and *L. reuteri*, have been found to influence social and emotional behaviors, alter expression of GABA receptors, and influence production of oxytocin, suggesting that certain *Lactobacillus* species may have therapeutic potential [20,101,102].

Some of the changes seen in less-represented taxa have the potential to disrupt gut homeostasis; for example, although many members of the *Enterobacteriaceae* Gram-negative family of bacteria are considered commensal, some members are pathogenic and have been associated with inflammatory bowel disease [103]. Other changes could be transient and less harmful; for example, expansion of *Akkermansia muciniphila* after LPS challenge is likely due to sickness behavior caused by inflammation, as this condition is known to cause this expansion [104]. However, overall, these findings point to how specific bacterial populations can potentially contribute to the loss of gut homeostasis.

We used cytokine concentrations as a comparative measure for the stability of immune responses of offspring and saw significant shifts in pro-inflammatory cytokines in the offspring from ABX treated dams, depending on group, age, and sex. Cytokines are key mediators of intercellular signaling and communication, as well as inflammatory responses. They are a diverse group of small proteins produced by cells, which lead to immune cell activation and production of additional pro- or anti-inflammatory cytokines, both of which are essential in influencing and regulating an immune response [105]. Pro-inflammatory cytokines can disrupt the microbiome and impact GVB function [27,106]. Significantly, cytokines must be tightly regulated in the brain for proper neurodevelopment and brain function [107]. Our findings warrant further research to determine if these shifts in brain cytokines due to maternal ABX lead to pathological outcomes.

We hypothesize that fewer changes persist at 6 months due to stabilization of the adult microbiota, while p21 microbiota is labile and more susceptible to large changes from early influences. However, when we look at predicted KEGG and MetaCyc pathways, we do see differences in the adults. Additionally, 6m have taxonomic differences not seen at p21. The functional capacity of the gut microbiota can give further insight into the effect maternal ABX had on the offspring. In our analysis, the ABX-cocktail adults had a different set of predicted metabolic pathways altered, mainly involving production of amino acids, and these were oppositely predicted based on age. Amino acid metabolism by the microbiota can shift bioavailability of amino acids for the host, increasing or decreasing them based on available substrate. Release of amino acids by the microbiota can also become precursors to other microbial molecules, including SCFA and neurotransmitters [108,109]. Amino acids are altered in ASD [110]; additionally, ASD microbiota transplanted into mice led to ASD-like behaviors associated with microbial metabolites, including altered amino acids [111,112]. Shifts in pathways noted in the vancomycin mice provide evidence that even moderate dysbiosis from maternal ABX is capable of significant changes in microbial metabolism that may ultimately have consequences for the host.

Despite a relatively small sample size, our study provides evidence of how antibiotics administered acutely during gestation can significantly influence offspring microbiota and immune outcomes. The maternal antibiotics were removed from drinking water immediately following birth to limit breastmilk transfer and any direct pup exposure. It is possible that there was carryover to breastmilk very early on. Behavioral analyses were outside the scope of this study and should be a focus of future studies. Behaviorally, we believe the best approach here would be to perform assays that are implicated in a number of neurodevelopmental disorders such as reciprocal social interactions, social approach, social preference, stereotyped and repetitive behaviors, fear, and anxiety. Additionally, due to sequencing limitations, we could not determine the exact species for all reads. These species were represented as ‘uncultured bacterium’ and some had multiple read variations detected which behave similarly for the most part. That is why we combined the different OTUs, for example *Muribaculaceae.* However, despite these limitations, we were still able to detect ABX-treatment, age, and even sex differences, thus increasing our understanding of the gut microbiota and the long-lasting differential effect maternal antibiotic use can have on the microbiota of male and female offspring.

## 5. Conclusions

Our results suggest that maternal ABX treatment leads to significantly altered gut bacterial communities, α-diversity, and β-diversity in offspring. Taxonomic variation because of maternal ABX differentially affects male and female offspring. This is highlighted in how the F/B ratios differ in females only. Maternal *ABX* alters CNS cytokine profile in offspring, resulting in decreased inflammatory cytokines, including IL-6, in the brain. Predicted metabolic pathways were increased in the antibiotic treatment groups, and this pathway influence was increased in male and female adult offspring. Together, these results further suggest that administration of ABX during late gestation and delivery could profoundly influence offspring microbiota and immune outcomes, with alterations that persist into adulthood. Future studies may disentangle the role of this altered gut microbiota on behavior outcomes, particularly those mentioned associated with ASD and how sex differences influence them.

## Data Availability

The data presented in this study are available on reasonable request from the corresponding author.

## Supplementary Materials

The following are available online at https://www.mdpi.com/article/10.3390/biomedicines10051042/s1, Figure S1: Prenatal ABX administration alters the guts microbiota diversity; Figure S2: Prenatal ABX administration alters offspring gut microbiota taxonomy; Figure S3: Taxonomy representation at genus and species level; Figure S4: Inducible cytokines are affected by ABX exposure; Figure S5: PICRUSt predictions of metabolic pathways; and Table S1: Percentage relative abundances of sequences classified to phylum.
