# Supplementary material for: Antibiotic Treatment during Pregnancy Alters Offspring Gut Microbiota in a Sex-Dependent Manner
Source: Biomedicines. 2022 Apr 30;10(5):1042. doi: 10.3390/biomedicines10051042 (PMC9138679; doi:10.3390/biomedicines10051042)
Supplement: Supplementary file 1 [file biomedicines-10-01042-s001.zip › biomedicines-1617504-supplementary.pdf]

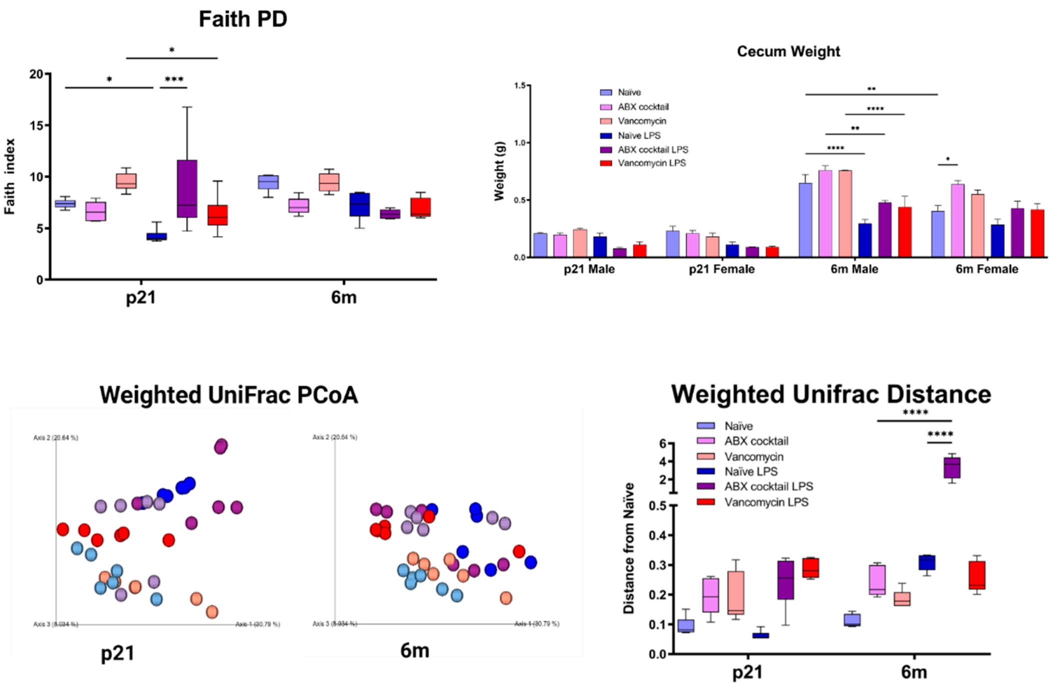

Figure S1. Prenatal ABX administration alters the guts microbiota diversity. Effect of ABX treatment and LPS challenge on cecum weight and male and females. α-diversity indexes using Faith PD index based on the Illumina sequencing data. β-diversity indexes using i) principal coordinate analysis (PCoA) plot and ii) box plots of distances based on Weighted UniFrac dissimilarity between samples. Each color represents a unique prenatal exposure baseline combined with or without stimulation: Naïve = light blue, Vancomycin = light red, ABX-cocktail = light purple, Naïve LPS = blue, Vancomycin LPS = red and ABX-cocktail LPS = purple. Naïve, vancomycin and ABX-cocktail samples were run in parallel, the number of mice in each group were male n=3 and female n=3. The levels of significance (p-values) determined by two-way ANOVA test followed by Tukey post-correction test for multiple comparisons. The values represent means + SEM, $^{*}p \leq 0.0332$, $^{**}p \leq 0.0021$, $^{***}p \leq 0.0002$ and $^{****}p \leq 0.0001$.

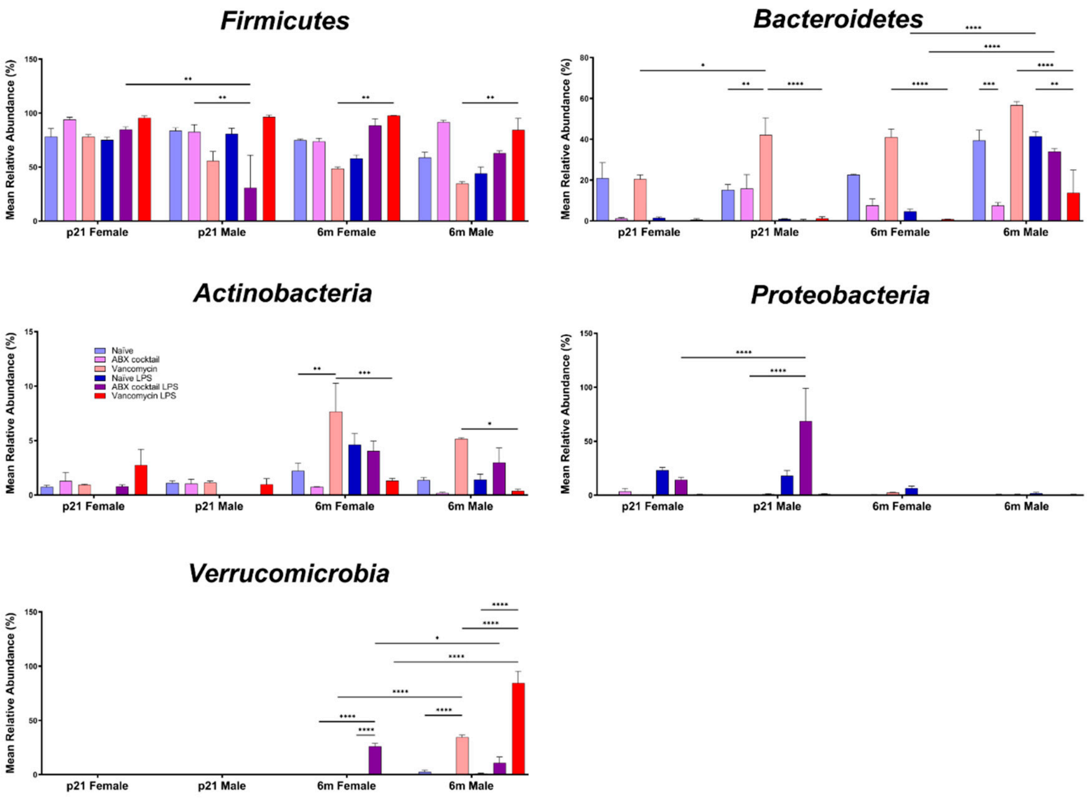

Figure S2. Prenatal ABX administration alters offspring gut microbiota taxonomy. (A, B) Relative abundances of sequences classified to phylum at p21 and 6m, respectively, with *Firmicutes* being the most abundant in both. Individual phyla compared to show significant differences between males and females and the treatment groups. Each color represents a unique prenatal basal exposure combined with or without LPS stimulation: Naïve = light blue, Vancomycin = light red, ABX-cocktail = light purple, Naïve LPS = blue, Vancomycin LPS = red and ABX-cocktail LPS = purple. Naïve, ABX-cocktail and Vancomycin samples were run in parallel, the number of mice in each group were male n=3 and female n=3. The levels of significance (p-values) determined by two-way ANOVA test followed by Tukey post-correction test for multiple comparisons. The bar values represent means + SEM, *p ≤ 0.0332, **p ≤ 0.0021, ***p ≤ 0.0002 and ****p ≤ 0.0001.

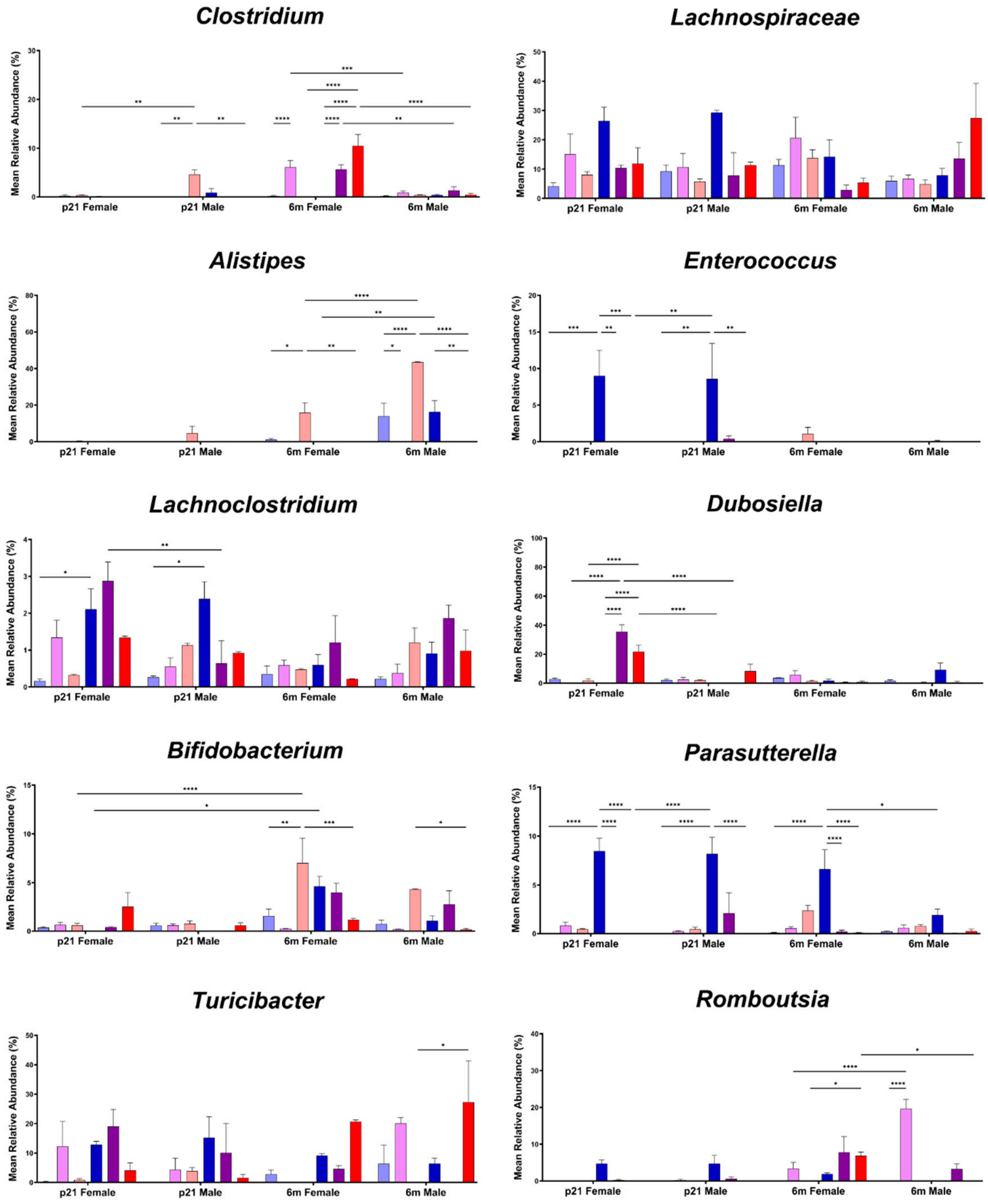

Figure S3. Taxonomy representation at genus and species level. (A, B) Relative abundances of sequences classified to genus at p21 and 6m, respectively. The remaining individual genera compared to show significant differences between males and females and the treatment groups. color represents a unique prenatal basal exposure combined with or without stimulation: Naïve = light blue, Vancomycin = light red, ABX-cocktail = light purple, Naïve LPS = blue, Vancomycin LPS = red and ABX-cocktail LPS = purple. Naïve, vancomycin and ABX-cocktail samples were run in parallel, the number of mice in each group were male n=3 and female n=3 . The levels of significance (p-values) determined by two-way ANOVA test followed by Tukey post-correction test for multiple comparisons. The bar values represent means + SEM, $^*p \leq 0.0332$, $^{**}p \leq 0.0021$, $^{***}p \leq 0.0002$ and $^{****}p \leq 0.0001$.

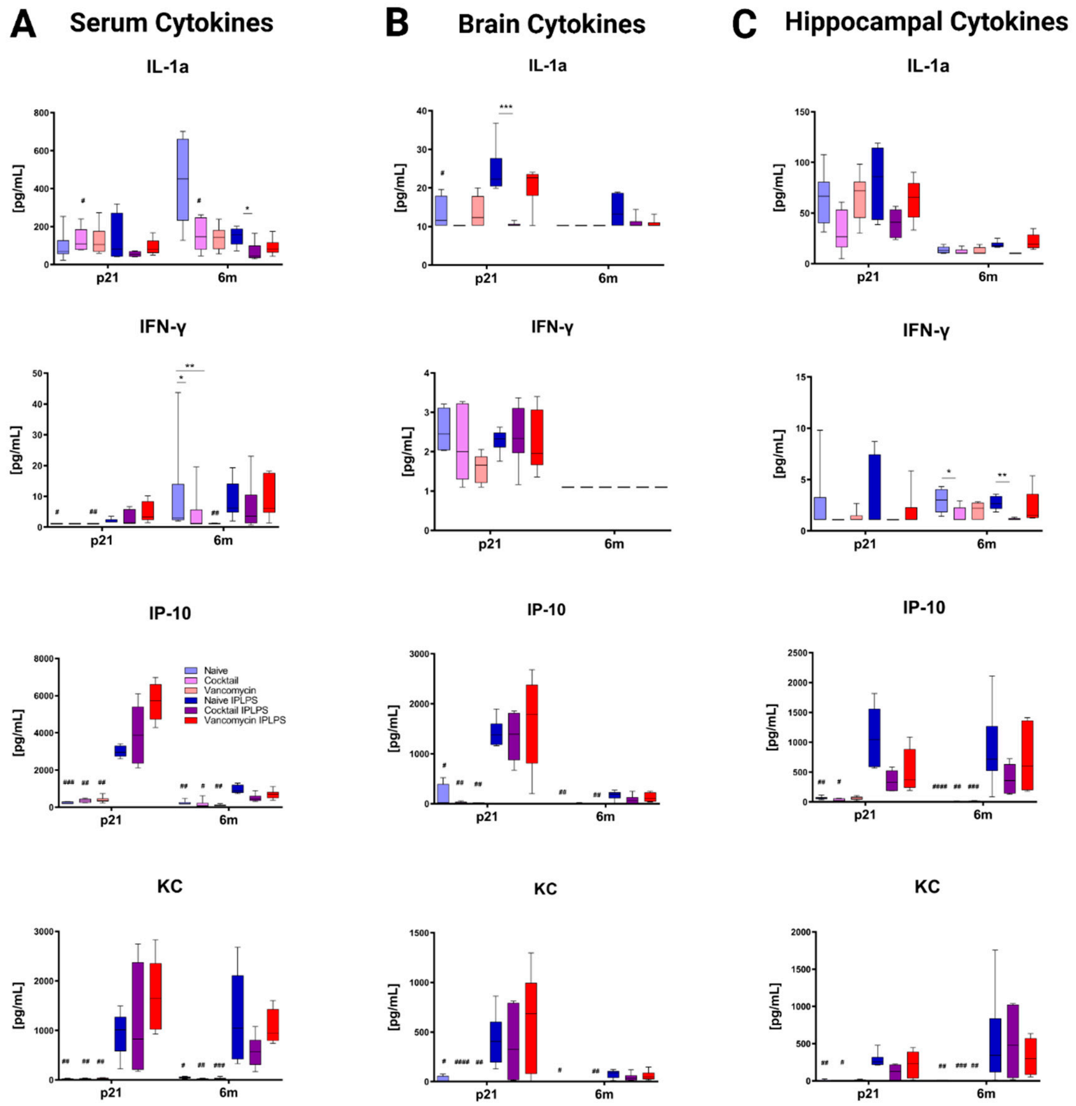

Figure S4. Inducible cytokines are affected by ABX exposure. Luminex data showing the responses of IL-1α, IFN-γ, IP-10, and KC from (A) serum (B) brain and (C) hippocampus. Each color represents a unique prenatal basal exposure with or without LPS stimulation: Naïve = light blue, Vancomycin = light red, ABX-cocktail = light purple, Naïve LPS = blue, Vancomycin LPS = red and ABX-cocktail LPS = purple. Naïve, vancomycin and ABX-cocktail samples were run in parallel. The levels of significance (p-values) determined by Kruskal-Wallis with Dunn's multiple comparison test. # is used to represent differences compare to LPS stimulation. Values represent median (min-max), *$p \leq 0.0332$, **$p \leq 0.0021$, ***$p \leq 0.0002$ and ****$p \leq 0.0001$.

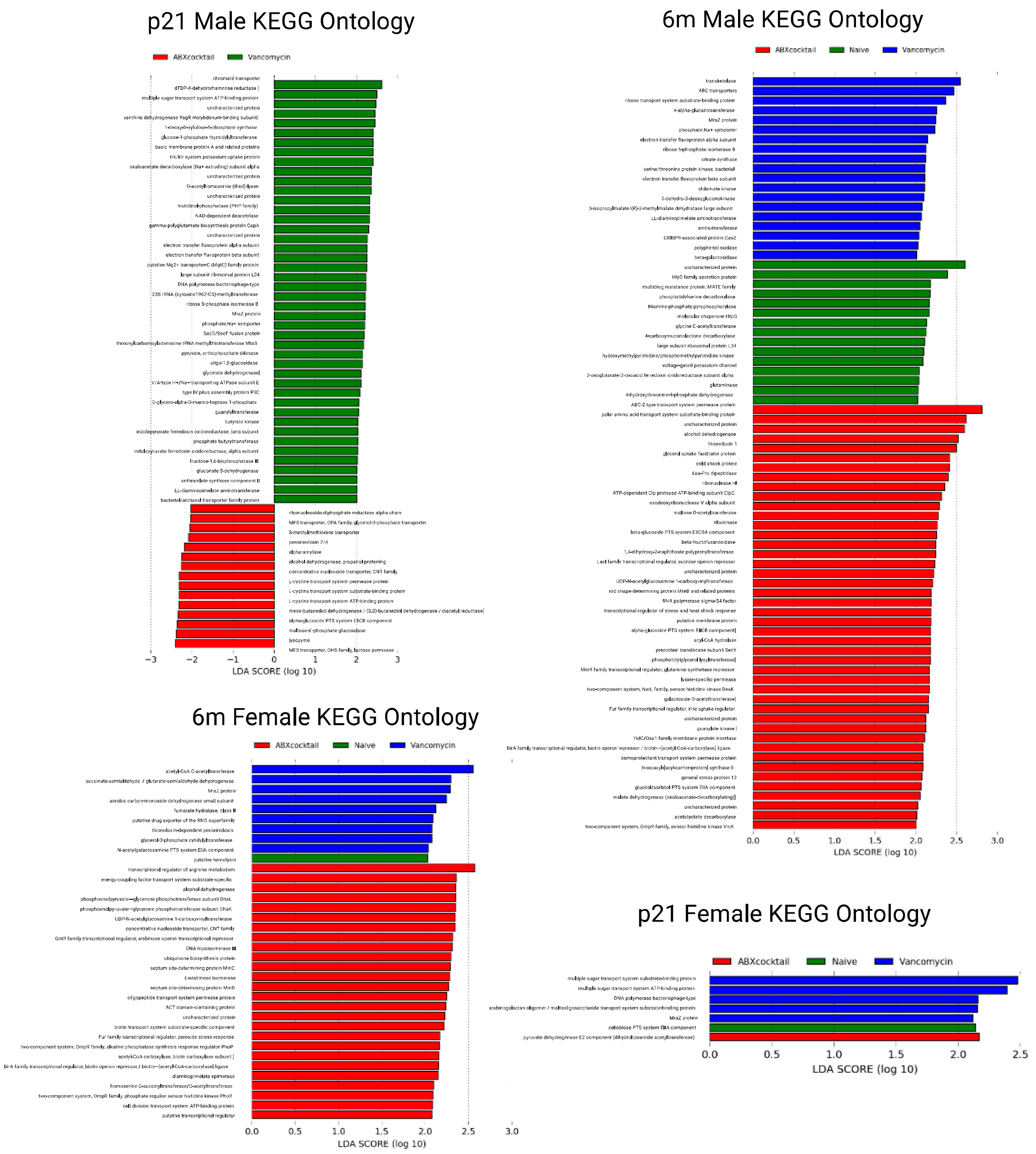

Figure S5. PICRUSt predictions of metabolic pathways. Functional summary for KEGG Ontology, Linear discriminant analysis (LDA) effect size (LEfSe) analysis revealed significant bacterial differences in gut microbiota between the antibiotic-treated groups and naïve group. from p21 male, p21 female, 6m male, and 6m female. LDA scores (log10) > 2 and P < 0.05 are shown. The number of mice in each group were male n=6 and female n=6.

Table S1. Percentage relative abundances of sequences classified to phylum at p21 and 6m, respectively. Individual phyla percentages compared to show differences between males and females and the treatment groups.

		Percentage Relative Abundance in samples					
		**Naïve**		**ABX-cocktail**		**Vancomycin**	
		**Male n=3**	**Female n=3**	**Male n=3**	**Female n=3**	**Male n=3**	**Female n=3**
**Phylum**							
Actinobacteria	p21	1	1	1	1	1	1
	6m	1	2	0	1	5	8
Bacteroidetes	p21	15	21	16	1	42	21
	6m	39	23	8	8	57	41
Firmicutes	p21	84	78	83	94	56	78
	6m	59	75	92	74	35	49
Proteobacteria	p21	0	0	0	4	1	0
	6m	0	0	1	1	1	3
Verrucomicrobia	p21	0	0	0	0	0	0
	6m	0	0	0	17	3	0
**Phylum post LPS**							
Actinobacteria	p21	0	0	0	1	1	3
	6m	1	5	3	4	0	1
Bacteroidetes	p21	1	2	0	0	1	1
	6m	41	5	34	0	14	1
Firmicutes	p21	81	75	31	85	97	96
	6m	44	58	63	89	84	98
Proteobacteria	p21	18	23	69	14	1	1
	6m	2	7	0	0	1	0
Verrucomicrobia	p21	0	0	0	0	0	0
	6m	11	26	0	7	1	0
